# The p53 status can influence the role of Sam68 in tumorigenesis

**DOI:** 10.18632/oncotarget.12305

**Published:** 2016-09-28

**Authors:** Naomi Li, Chau Tuan-Anh Ngo, Olga Aleynikova, Nicole Beauchemin, Stéphane Richard

**Affiliations:** ^1^ Terry Fox Molecular Oncology Group and the Bloomfield Center for Research on Aging, Sir Mortimer B Davis Jewish General Hospital, Lady Davis Institute for Medical Research, Montréal, Québec H3T 1E2, Canada; ^2^ Department of Medicine and Oncology, McGill University, Montréal, Québec H3A 1A1, Canada; ^3^ Department of Pathology, Jewish General Hospital, Montréal, Québec H3T 1E2, Canada; ^4^ Department of Biochemistry, McGill University, Montréal, Québec H3G 1Y6, Canada; ^5^ Rosalind and Morris Goodman Cancer Centre, Montréal, Québec H3A 1A3, Canada

**Keywords:** Sam68, RNA binding protein, p53, tumorigenesis, mouse models

## Abstract

The expression and activities of RNA binding proteins are frequently dysregulated in human cancer. Their roles, however, appears to be complex, with reports indicating both pro-tumorigenic and tumor suppressive functions. Here we show, using two classical mouse cancer models, that the role of KH-type RNA binding protein, Sam68, in tumor development can be influenced by the status of the p53 tumor suppressor. We demonstrate that in mice expressing wild type p53, Sam68-deficiency resulted in a higher incidence and malignancy of carcinogen-induced tumors, suggesting a tumor suppressive role for Sam68. In marked contrast, Sam68-haploinsufficiency significantly delayed the onset of tumors in mice lacking p53 and prolonged their survival, indicating that Sam68 accelerates the development of p53-deficient tumors. These findings provide considerable insight into a previously unknown relationship between Sam68 and the p53 tumor suppressor in tumorigenesis.

## INTRODUCTION

Src-associated in mitosis of 68 kDa (Sam68), also known as KHDRBS1, is a KH-type RNA binding protein of the signal transduction and activation of RNA metabolism (STAR) family [1–3]. The expression of Sam68 is often elevated in human cancers, including breast, colorectal, esophageal, endometrial, cervical, renal, lung, bladder, ovarian, neural, and prostate cancers [4–15]. Furthermore, overexpression of Sam68 has been shown to correlate with poor survival prognosis in renal cell carcinoma, colorectal cancer, and non-small cell lung cancer patients [5, 9, 10]. In some cancers, high expression of Sam68 correlates with low expression of certain miRNAs shown to target Sam68, such as in the case of miR-203 and miR-204 observed in neuroblastoma and breast cancer, respectively [13, 16]. Collectively, these findings suggest that Sam68 has a positive role in tumor progression.

To further investigate the physiological roles of Sam68, a mouse model was generated by homologous recombination [17]. These whole body knockout mice are born with no visible deformities and live to old age [17]. Phenotypically, Sam68-null mice are lean and are protected against age-related bone loss [17, 18]. These mice also have significant motor coordination defects attributed to RNA metabolism dysregulation in neurons [19–22]. Interestingly, crossbreeding with spinal muscular atrophy (SMAΔ7) mice rescued the body weight and viability of SMAΔ7, suggesting that the loss of Sam68 could be a therapeutic avenue for some patients with spinal muscular atrophy [23]. Sam68-null mice do not develop spontaneous tumors, demonstrating that Sam68 is not a *bona fide* tumor suppressor like p53 [24, 25]. However, Sam68-haploinsufficiency delays the onset of MMTV-PyMT-driven mammary tumors and reduces dissemination of lung metastasis, implicating that Sam68 is required for mammary tumorigenesis [26].

As a prototypic member of the STAR proteins, Sam68 has signaling properties and functions downstream of Src family kinases to promote cell survival [26, 27]. In addition, Sam68 regulates cell cycle progression and apoptosis. Specifically, Sam68 modulates transcription and mRNA translation, and functions as a major regulator of pre-mRNA alternative splicing [2]. It has been demonstrated that Sam68 depletion by siRNA promotes cell cycle arrest, and decreases the proliferation of many cancer cell lines [4, 7, 8, 15]. In particular, Sam68 has been shown to enhance the inclusion of CD44 variable exon 5 and the splicing of cyclin D1b isoform [28, 29], both of which are known to promote cell proliferation. Depletion of Sam68 from breast cancer cell lines upregulates cyclin-dependent kinase inhibitors p21 and p27, while also reducing Akt phosphorylation [4]. Moreover, Sam68 contributes to the protection of prostate cancer cells from genotoxic agents [15]. Hence, these findings strongly suggest Sam68 as an RNA binding protein required for the proliferation of certain cancer cells.

Sam68 has also been shown to harbor tumor suppressive functions, but this activity is not well understood, nor is there any evidence of this tumor suppressive role *in vivo*. It has been proposed that the loss of Sam68 leads to NIH3T3 cell transformation; however, this was not rescued by Sam68, suggesting that additional events were responsible for transformation in this report [30]. Consistent with its tumor suppressor-like activities, the overexpression of Sam68 in murine fibroblasts induces cell cycle arrest and apoptosis [31]. Moreover, Sam68 expression favors alternative splicing of the pro-apoptotic Bcl-x(s) isoform, which further demonstrates its tumor suppressor-like activity [32, 33].

The development of mouse cancer models has greatly advanced our understanding of the mechanisms underlying tumor initiation, progression, metastasis, and acquired chemoresistance [34–36]. Specifically, the chemical carcinogen-based inducible tumor models are essential tools for investigating tumor development in a controlled manner [34, 35]. For instance, the azoxymethane (AOM)-inducible mouse model provides a reproducible system for studying spontaneous colon cancer, where tumors develop frequently in the distal part of the colon, and exhibit features similar to spontaneous colon cancer found in humans [37]. Additionally, genetically engineered mice, such as the p53-deficient mice [38], have been particularly valuable for the identification of molecular mechanisms and pathways affected by p53-deficiency, as the loss or mutation of *TP53* occurs in approximately 50% of all cancers [25, 39]. Indeed, p53-deficient mice are predisposed to a range of spontaneous tumors, including lymphomas and sarcomas [40, 41]. Recently, we have identified Sam68 as a transcriptional co-activator of p53 [42], suggesting that Sam68 may also have tumor suppressor activities like p53.

In this study, we report the role of Sam68 in tumorigenesis using both p53-proficient and p53-deficient mouse models of cancer. We show that in p53 wild type mice, Sam68-deficiency enhanced AOM-induced colon tumorigenesis, whereas in p53-deficient mice, Sam68-haploinsufficiency delayed the onset of spontaneous tumors. Hence, we demonstrate for the first time that p53 status can influence the role of Sam68 during tumorigenesis.

## RESULTS

### Sam68-deficiency increases AOM-induced tumor burden and malignancy in p53 wild type mice

**Figure 1:**
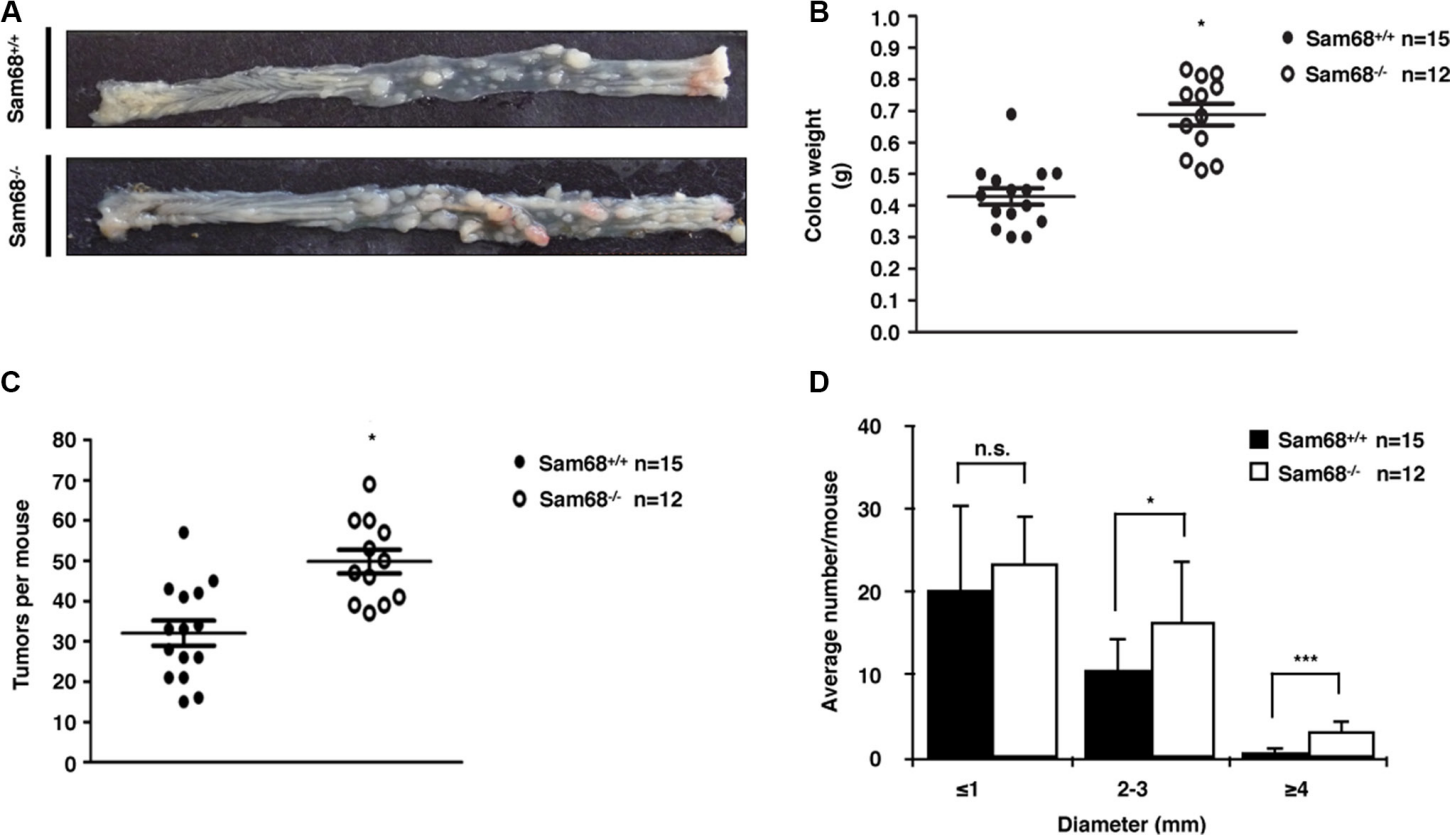
Sam68-deficent mice showed higher AOM-induced tumor burden and size (**A**) Colons isolated from p53^+/+^;Sam68^+/+^ and p53^+/+^;Sam68^−/−^ FVB mice after AOM treatment. (**B**) Weight of colons isolated from p53^+/+^;Sam68^+/+^ and p53^+/+^;Sam68^−/−^ mice after AOM treatment. (**C**–**D**) Quantification of AOM-induced colon tumor numbers and average tumor size in p53^+/+^;Sam68^+/+^ and p53^+/+^;Sam68^−/−^ mice. All error bars represent S.D. of the mean (*p ≤ 0.05, **p ≤ 0.005, ***p ≤ 0.0005).

**Figure 2:**
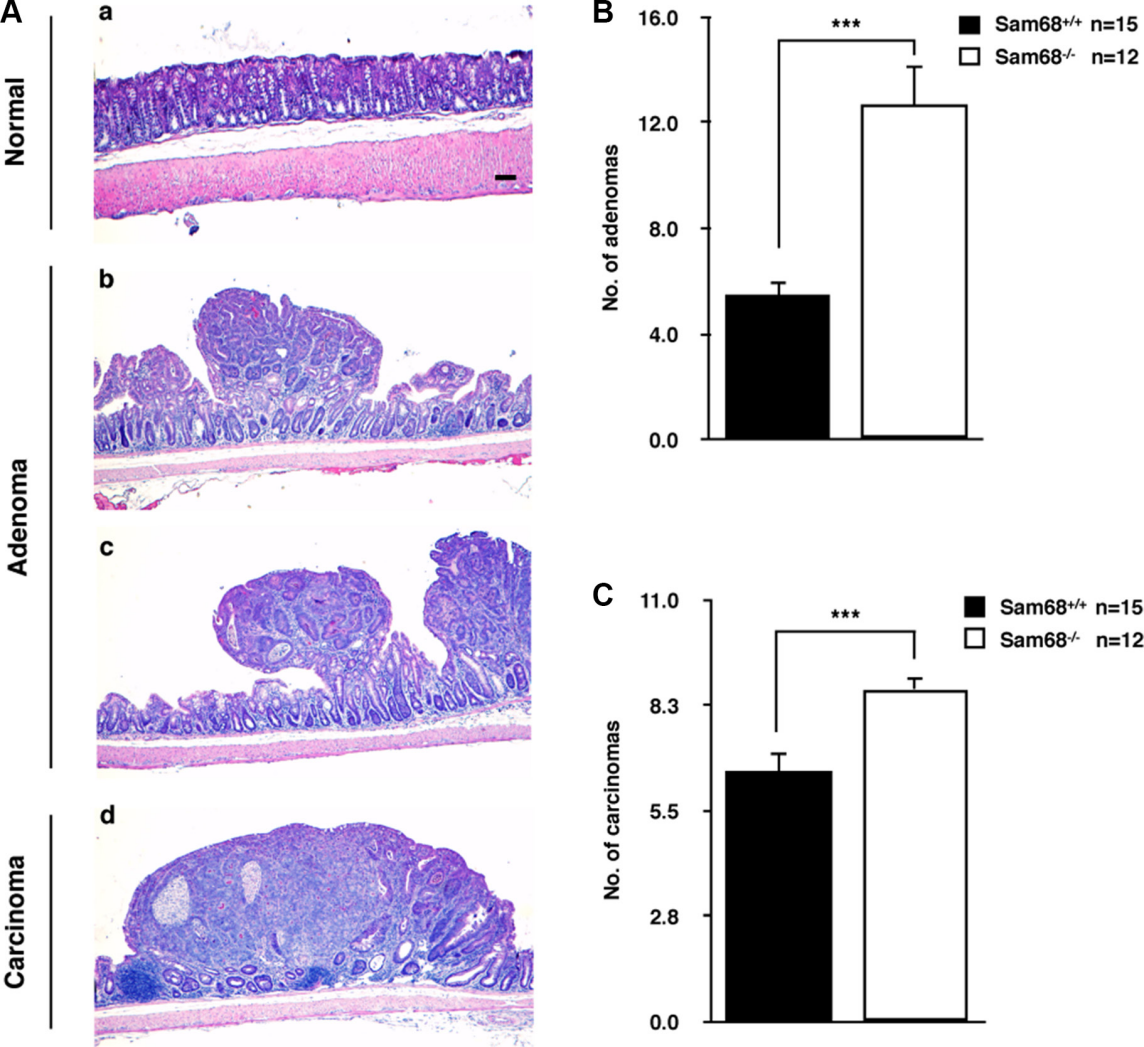
Sam68-deficient mice developed colon tumors with increased malignancy (**A**) Representative H&E stains of untreated normal colon (a), adenoma from AOM-treated p53^+/+^;Sam68^+/+^ mice (b) and p53^+/+^;Sam68^−/−^ mice (c), and carcinoma from AOM-treated p53^+/+^;Sam68^−/−^ mice. Scale bar, 100μm. (**B**–**C**) Quantification of adenomas and carcinomas from colons of AOM-treated p53^+/+^;Sam68^+/+^ and p53^+/+^;Sam68^−/−^ mice. All error bars represent S.D. of the mean (*p ≤ 0.05, **p ≤ 0.005, ***p ≤ 0.0005).

**Figure 3:**
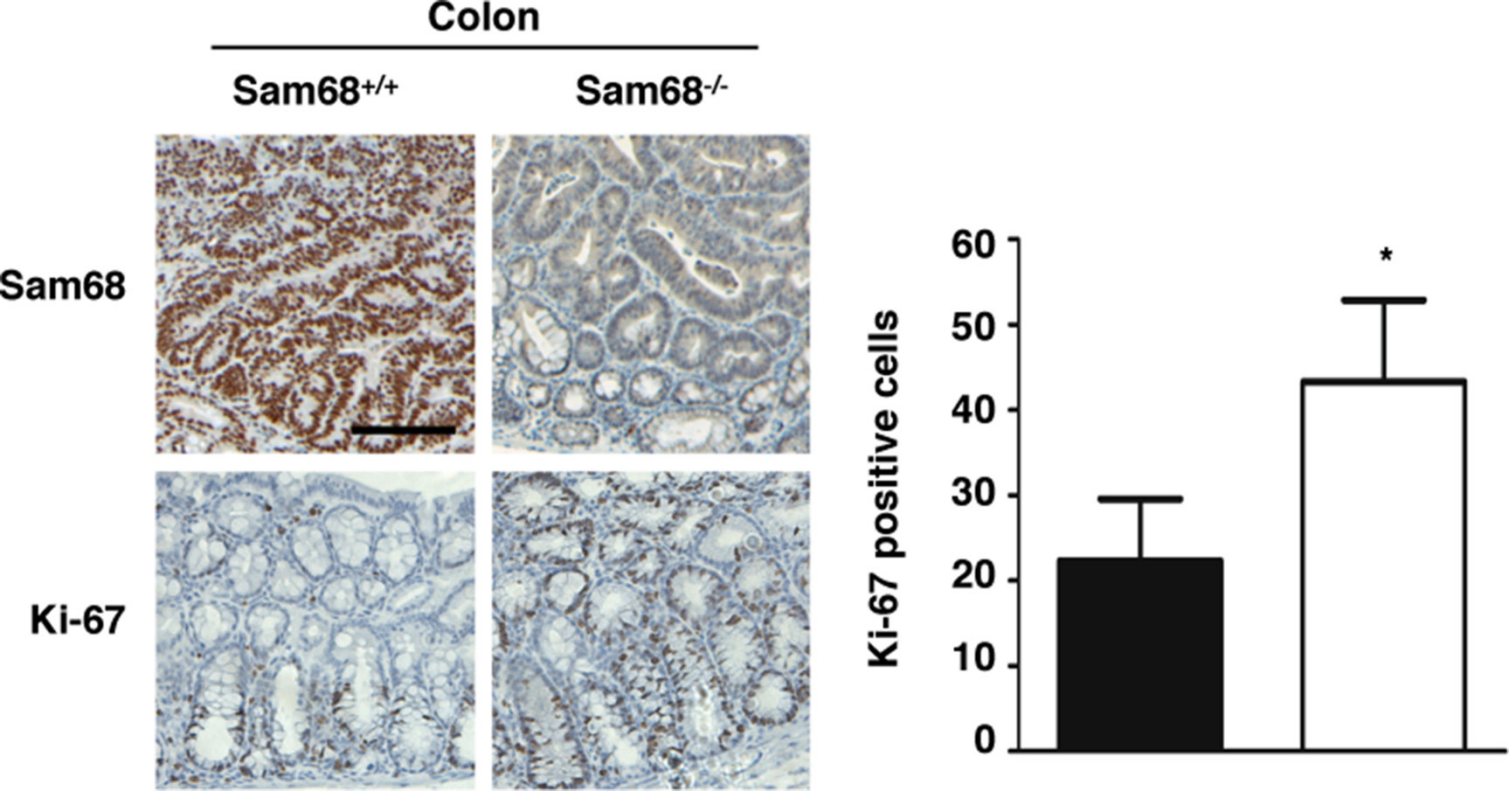
Sam68-deficient mouse colons contain more Ki-67-positive cells Immunohistochemistry analysis of Sam68 and Ki-67 in colons isolated from AOM-treated p53^+/+^;Sam68^+/+^ and p53^+/+^;Sam68^−/−^ mice. Scale bar, 100 μm. All error bars represent S.D. of the mean (*p ≤ 0.05, **p ≤ 0.005, ***p ≤ 0.0005).

To investigate the role of Sam68 in tumor development where p53 is wild type, we chose a carcinogen-based mouse cancer model [34, 40]. Specifically, we used an azoxymethane (AOM)-inducible mouse model of colon carcinogenesis. AOM exerts colonotropic carcinogenicity in a non-specific manner by alkylating DNA to drive spontaneous tumor initiation [37]. Importantly, p53 is not generally mutated in AOM-induced lesions [43], allowing us to study the effect of Sam68 in this cancer model. Cohorts of fifteen p53^+/+^; Sam68^+/+^ and p53^+/+^; Sam68^−/−^ mice, backcrossed 7 generations in the FVB background, were treated with AOM for 8 weeks and examined each week for 13 weeks. Three of the p53^+/+^; Sam68^−/−^ mice were sacrificed early due to acute clinical features. In the remaining p53^+/+^; Sam68^−/−^ mice, as well as the p53^+/+^; Sam68^+/+^ mice, aberrant growths were restricted to the colon with no macroscopically visible metastasis. The p53^+/+^; Sam68^−/−^ mice had a statistically significant increase in colon weight (0.69 g ± 0.03 versus 0.43 g ± 0.03) because of the higher tumor burden (Figure 1A and 1B). Moreover, p53^+/+^;Sam68^−/−^ mice displayed increased number of tumors per colon (49.83 ± 2.93 versus 32.07 ± 3.07; Figure 1C), and generally developed larger tumors (2–3 mm, ≥ 4 mm) compared to the p53^+/+^;Sam68^+/+^ mice (Figure 1D). Further examinations revealed that AOM-treated p53^+/+^; Sam68^−/−^ mice had a statistically significant increase in the number of adenomas (12.67 ± 1.45 versus 5.5 ± 0.5) and carcinomas (8.67 ± 0.33 versus 6.5 ± 0.5) compared to p53^+/+^;Sam68^+/+^ mice (Figure 2). Concomitantly, p53^+/+^;Sam68^−/−^ mice showed a marked increase in the cell proliferation marker Ki-67 compared to p53^+/+^;Sam68^+/+^ mice (45.77 ± 2.43 versus 23.07 ± 2 per crypt; Figure 3). Taken together, p53^+/+^;Sam68^−/−^ mice exhibited a higher incidence of AOM-induced colon cancer, suggesting a crucial function for Sam68 in suppressing the malignant progression of these tumors.

### Sam68-haploinsufficiency delayed tumor onset and prolonged survival in p53-deficient mice

**Figure 4:**
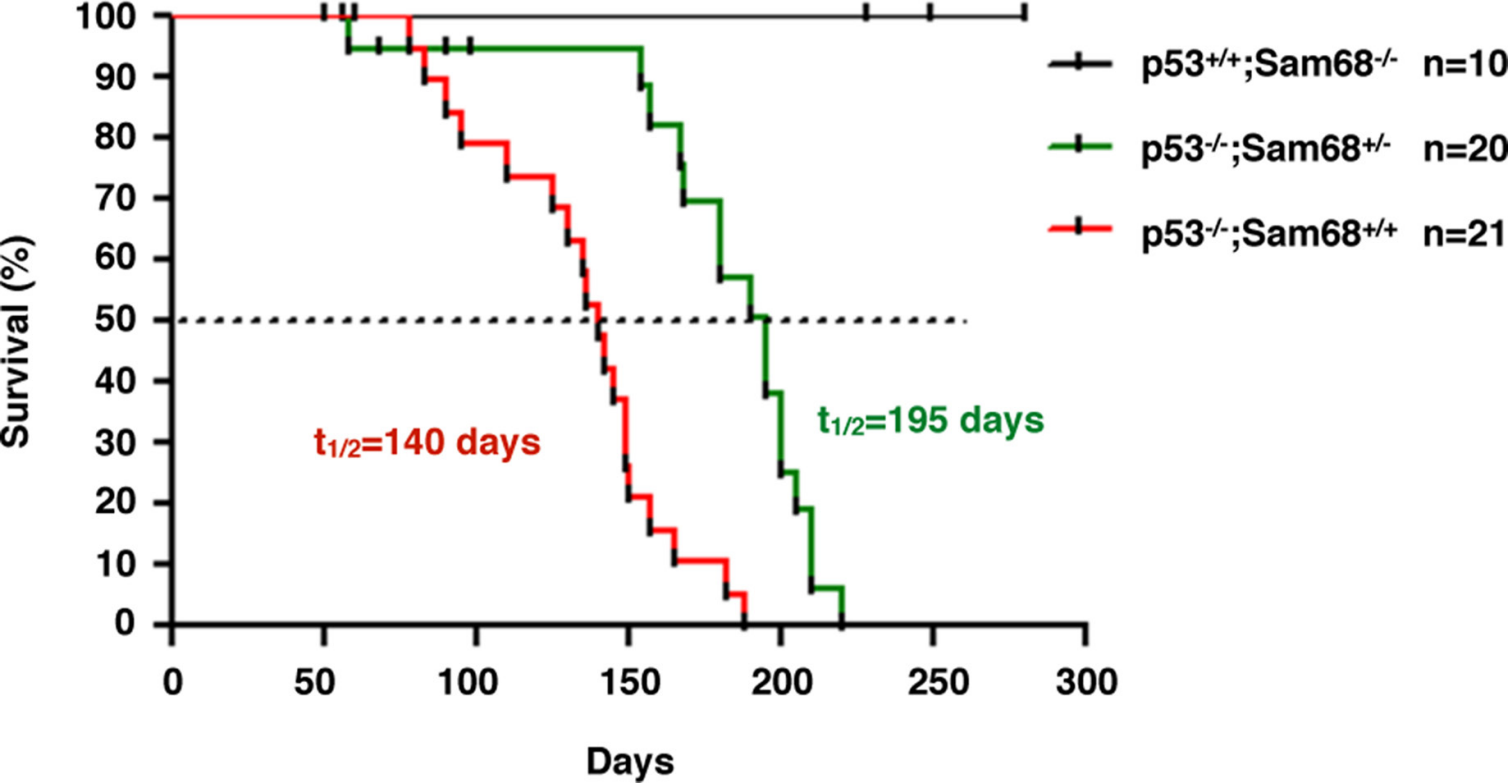
Sam68-haploinsufficiency delayed tumor onset in p53-deficient mice Kaplan-Meier survival curve of tumor incidence in p53^+/+^; Sam68^−/−^, p53^−/−^; Sam68^+/+^, and p53^−/−^; Sam68^+/−^ C57BL/6 mice. Tumor formation was monitored over a period of 10 months, and sacrificed at first clinical signs of discomfort (*p* < 0.0001).

We next examined whether the absence of Sam68 affects the onset of spontaneous tumors in p53-null mice [38, 40], given that we have recently reported Sam68 as a transcriptional co-activator of p53 [42]. We initially crossbred Sam68^−/−^ (> 14 generations in C57BL/6 background) with p53^−/−^ mice, but the breeding was complicated by the fact that Sam68 homozygous females have impaired fertility and males are infertile [26, 44]. For these reasons, only Sam68 heterozygotes were used for breeding, which are known to express lower levels of Sam68 than wild type mice [26]. Additionally, few p53^−/−^ females were obtained, therefore the breeding was performed with p53^−/−^; Sam68^+/−^ males and p53^+/−^;Sam68^+/−^ females. Few p53^−/−^;Sam68^−/−^ mice were obtained for unknown reasons but they did survive into adulthood. Thus, we compared tumor latency between p53^−/−^;Sam68^+/+^ and p53^−/−^;Sam68^+/−^ mice. We observed that Sam68-haploinsufficiency significantly delayed tumor onset in p53-deficient mice from 140 days (p53^−/−^;Sam68^+/+^ mice, *n* = 21) to 195 days (p53^−/−^;Sam68^+/−^ mice, *n* = 20, *p* < 0.0001; Figure 4). Like p53^−/−^;Sam68^+/+^ mice, p53^−/−^;Sam68^+/−^ mice succumbed to mostly lymphomas and sarcomas [38]. Consistent with previous studies, none of the control p53^+/+^;Sam68^−/−^ mice (*n* = 10) developed tumors in the 300 days of the experiment (Figure 4) [17]. Altogether, our findings suggest that Sam68 has pro-tumorigenic properties in the absence of p53.

## DISCUSSION

In the present study, we examined the physiological role of Sam68 in tumor development using two different mouse cancer models. For Sam68-proficient and deficient mice in a p53 wild type FVB background, we implemented the azoxymethane (AOM)-inducible colon carcinogenesis model. We observed that Sam68-deficient (p53^+/+^;Sam68^−/−^) mice displayed higher tumor burden with increased malignancy than wild type controls (p53^+/+^;Sam68^+/+^), demonstrating that Sam68 has tumor suppressor-like activities *in vivo*. We also generated p53-null mice expressing Sam68 (p53^−/−^;Sam68^+/+^) or haploinsufficient dose of Sam68 (p53^−/−^;Sam68^+/−^). In this p53^−/−^ C57BL/6 background, we observed that Sam68 haploinsufficiency caused significant delays in the onset of spontaneous tumors compared to Sam68 wild type mice. These findings show that in the absence of p53, Sam68 can exhibit pro-tumorigenic properties. Collectively, our results demonstrate that p53 seems to act as a switch to influence whether Sam68 slows or accelerates tumor development. Taken together, these findings provide insight into a previously unknown relation between Sam68 and the p53 tumor suppressor in tumorigenesis.

RNA binding proteins are becoming increasingly appreciated as fundamental players in human cancers [45, 46]. Like tumor suppressors, abnormal expression or mutations in RNA binding proteins have been shown to alter their function, and many are associated with a cancer phenotype [46]. We posit for the first time that Sam68-deficiency leads to increased tumorigenesis, where p53^+/+^;Sam68^−/−^ mice displayed significantly higher AOM-induced tumor burden and malignancy compared to p53^+/+^;Sam68^+/+^ mice. Therefore, in this context, Sam68 behaves as a tumor suppressor. Indeed, Sam68 has been shown to be pro-apoptotic mainly by regulating Bcl-x(s) splicing [31–33]. Moreover, the fact that Sam68 functions as a co-activator of p53 is consistent with its tumor suppressor role [42]. Interestingly, it has been shown that p53 does not influence the rate of tumor initiation, but instead is required to prevent the malignant progression of tumors [47], which parallels the phenotype of the AOM mouse model. However, untreated Sam68^−/−^ mice are not predisposed to spontaneous tumor development [17], similar to the phenotypes of p21^−/−^ and p21^−/−^;Puma^−/−^;Noxa^−/−^ mice [48, 49]. This perhaps explains why, as observed with the *p21* gene, that no known mutations of *Sam68* have been found in human tumors [50]. Furthermore, unlike p53, the function of Sam68 may be partially redundant with other STAR family RNA binding proteins, such as Sam68 paralogs SLM-1 and SLM-2 [51, 52].

Given the interplay between Sam68 and p53, it is almost expected that Sam68 would lose its tumor suppressive function when p53 is depleted. Indeed, studies in p53^−/−^ mice showed that decreased Sam68 expression significantly delayed the onset of spontaneous tumors, indicating a pro-tumorigenic role for Sam68 in this mouse model. Fu *et al.* recently reported that Sam68 lessens colon tumor development in Apc^min716/+^ mice via regulation of the PARP1-NF-κB-anti-apoptotic gene axis [53]. Furthermore, Sam68-deficient human colon cancer cells are sensitive to genotoxic stress-induced apoptosis [53].

Although it is uncertain how p53 loss or inactivation can contribute to the pro-tumorigenic function of Sam68, a large body of literature has elucidated molecular mechanisms by which Sam68 can support tumor growth. As a STAR family protein, Sam68 can link signal transduction with RNA processing to modulate cancer-relevant splicing events that enhance cell survival and proliferation [1–3]. For instance, Fyn-mediated tyrosine phosphorylation of Sam68 promotes splicing of the anti-apoptotic Bcl-x(L) isoform in prostate and pancreatic cancer cells [32, 54]. Additionally, Sam68 has been identified as a downstream target of the MAPK ERK1/2 pathway, where serine/threonine phosphorylation of Sam68 enhances tumorigenic CD44 variable exon 5 inclusion [28]. Similarly, activation of the MAPK pathway also stimulates Sam68-mediated splicing of cyclin D1b isoform to favor cell proliferation [29]. The involvement of Sam68 in signaling during tumorigenesis has also been observed previously in a MMTV-PyMT mouse model, where Sam68-haploinsufficiency delayed mammary tumorigenesis [26], consistent with the phenotype of our p53^−/−^;Sam68^+/−^ mice. Of note, mammary tumor cells derived from the MMTV-PyMT mice are p53 wild type and are sensitive to p53 gene therapy, but the antitumor mechanisms remain elusive [55]. Furthermore, Sam68 may promote tumorigenesis by taking part in the transcription machinery. In prostate cancer cells, Sam68 functions as a transcriptional co-activator of the androgen receptor (AR) [14], a nuclear hormone receptor driving the onset and progression of prostate cancer [56]. Sam68 also modulates the promoter specificity of NF-κB to induce CD25 expression implicated in tumorigenesis [57]. Additionally, Sam68 is an essential component of the MLL oncogenic transcriptional complex by acting as a bridging molecule for MLL and PRMT1, and the knockdown of Sam68 suppressed MLL-mediated transformation [58]. In further support of its pro-tumorigenic properties, Sam68 overexpression may drive tumor progression by downregulating tumor suppressive miRNAs, including miR-203 and miR-204 [13, 16].

Sam68 overexpression, phosphorylation, and its cytoplasmic localization have been associated with a significant risk factor for poor prognosis [8, 9, 59]. It is also known that extensive post-translational modifications such as phosphorylation can influence its RNA binding activity [28, 32, 60]. For example, the elevated expression, mislocalization, and tyrosine phosphorylation of Sam68 all contribute to poor prognosis in breast cancer patients [4, 59]. Consistently, aberrant tyrosine phosphorylation and cytoplasmic localization of Sam68 has also been observed in various other cancer cells [8, 9, 26, 61, 62]. Together, the evidence suggests that aberrant Sam68 regulation, including sequestration of Sam68 from its nuclear role or inactivation by phosphorylation contributes to exacerbate tumorigenesis.

Although further studies are required to understand the mechanism underlying p53's ability to switch the functional outcome of Sam68, we have illustrated the importance of defining the molecular contexts in which targeting RNA binding proteins may be beneficial for anticancer therapy. Much remains to be defined on how RNA binding proteins and their regulatory networks contribute to tumor initiation and progression.

## MATERIALS AND METHODS

### Mice breeding, AOM treatment, and colon tumor preparations

All animal procedures followed the Canadian Council on Animal Care guidelines and were approved by the McGill Animal Care Committee. Age-matched (10 to 12 weeks) p53^+/+^;Sam68^+/+^ and p53^+/+^; Sam68^−/−^ mice in FVB background (cohorts of 15 in each group) were injected intraperitoneally once a week for 8 weeks with 10 mg/kg of body weight azoxymethane (AOM) (Sigma-Aldrich) dissolved in saline. Animals were systematically examined twice per week for the appearance of clinical symptoms. Animals showing signs of discomfort such as weight loss, rectal bleed, and prolapse were sacrificed. End points were defined as 13 weeks after the first injection. After sacrificing the mice, entire colons were removed, rinsed with PBS, opened longitudinally and fixed flat on strips of 4% paraformaldehyde-soaked Whatman filter paper. Colons were assessed in a blinded fashion under a stereo-dissecting microscope as previously described [63]. Tumors were measured using a clear transparency of 1 mm^2^ graph paper, and the total surface area was determined based on the total number of squares overlaying the tumor, as described [64].

The p53^−/−^ mice (Catalog# 002101) were obtained from JAX laboratories. Both Sam68^−/−^ and p53^−/−^ mice were maintained on a C57BL/6 background and crossbred. Animals showing signs of discomfort such as weight loss were sacrificed.

### Mouse genotyping

Total genomic DNA was extracted from mouse earpiece using AccuStart II Mouse Genotyping Kit (Quanta) following the manufacturer's protocol. Genomic PCR was performed with the following primers: *Sam68* (forward 1) 5′- GAT ATG ATG GAT GAT ATC TTG TCA G-3′; (forward 2) 5′- CTA AAG CGC ATG CTC CAG A-3′; (reverse) 5′- AAA TCC TAA CCC TCC TCA GTC A-3′; *p53* (forward) 5′- ACA GCG TGG TGG TAC CTT AT-3′; (reverse 1) 5′-TAT ACT CAG AGC CGG CCT-3′; (reverse 2) 5′-CTA TCA GGA CAT AGC GTT GG-3′. PCR products were analyzed on 1.5% agarose gel stained with ethidium-bromide.

### H&E staining and immunohistochemistry

Hematoxylin and eosin (H&E) or immunohistochemistry staining were performed on p53^+/+^; Sam68^+/+^ and p53^+/+^; Sam68^−/−^ mice colons at the IRIC (Université de Montréal). Quantitative assessment was done as previously described [65].

### Statistical analysis

Statistical analysis was performed using Student's *t* test with the GraphPad software. A *p*-value of < 0.05 was considered to be statistically significant.
